# Breaking the ‘Undruggable’ Barrier: Anti-PD-1/PD-L1 Immunotherapy for Non-Small Cell Lung Cancer Patients with *KRAS* Mutations—A Comprehensive Review and Description of Single Site Experience

**DOI:** 10.3390/cancers15143732

**Published:** 2023-07-23

**Authors:** Izabela Chmielewska, Paweł Krawczyk, Anna Grenda, Magdalena Wójcik-Superczyńska, Natalia Krzyżanowska, Michał Gil, Janusz Milanowski

**Affiliations:** Department of Pneumonology, Oncology and Allergology, Medical University of Lublin, 20-090 Lublin, Poland; izabela.chmielewska@umlub.pl (I.C.); pawel.krawczyk@umlub.pl (P.K.); magvoj@poczta.onet.pl (M.W.-S.); natalia.krzyzanowska@umlub.pl (N.K.); michal.gil@umlub.pl (M.G.); janusz.milanowski@umlub.pl (J.M.)

**Keywords:** *KRAS* gene, PD-L1, immunotherapy, non-small cell lung cancer, sotorasib, adagrasib

## Abstract

**Simple Summary:**

*KRAS* gene mutations are among the most common oncogenic lesions in NSCLC patients. For many years, they were considered “incurable”. This is now changing and therapeutic options are available for NSCLC patients with mutated *KRAS*. It is likely that the effectiveness of immunotherapy in *KRAS^mut^* NSCLC patients does not depend only on the presence of druggable lesions in this gene, but also on the molecular background—co-mutations in the *STK11*, *KEAP1* and *TP53* genes. This article reviews the literature on the efficacy of immunotherapy in NSCLC patients with *KRAS* mutation. It also presents our own experience with the use of immunotherapy in patients with *KRAS^mut^* NSCLC.

**Abstract:**

*Kirsten rat sarcoma viral oncogene homologue* (*KRAS*) gene mutations are among the most commonly found oncogenic alterations in non-small cell lung cancer (NSCLC) patients. Unfortunately, *KRAS* mutations have been considered “undruggable” for many years, making treatment options very limited. Immunotherapy targeting programmed death-ligand 1 (PD-L1), programmed death 1 (PD-1) and cytotoxic T lymphocyte antigen 4 (CTLA-4) has emerged as a promising therapeutic option for NSCLC patients. However, some studies have suggested a lower response rate to immunotherapy in KRAS-mutated NSCLC patients with the coexistence of mutations in the *STK11* (*Serine*/*Threonine Kinase 11*) gene. However, recent clinical trials have shown promising results with the combination of immunotherapy and chemotherapy or immunotherapy and KRAS inhibitors (sotorasib, adagrasib) in such patients. In other studies, the high efficacy of immunotherapy has been demonstrated in NSCLC patients with mutations in the *KRAS* gene that do not coexist with other mutations or coexist with the *TP53* gene mutations. In this paper, we review the available literature on the efficacy of immunotherapy in *KRAS*-mutated NSCLC patients. In addition, we presented single-site experience on the efficacy of immunotherapy in NSCLC patients with *KRAS* mutations. The effectiveness of chemoimmunotherapy or immunotherapy as well as KRAS inhibitors extends the overall survival of advanced NSCLC patients with the G12C mutation in the *KRAS* gene to 2–3 years. This type of management has become the new standard in the treatment of NSCLC patients. Further studies are needed to clarify the potential benefits of immunotherapy in *KRAS*-mutated NSCLC patients and to identify potential biomarkers that may help predict response to therapy.

## 1. Introduction

Lung cancer (LC), responsible for a significant number of cancer-related deaths worldwide, is estimated to cause approximately 2.20 million new cases (65% of them are men, and 35% are women) and 1.79 million deaths annually. LC is one of the most frequently diagnosed cancers and remains the leading cause of all cancer-related deaths [1,2]. The main risk factor for lung cancer is cigarette smoking, with 60–90% of LC patients being current or former smokers. The age of patients at the moment of LC diagnosis is 65–70 years [2,3].

Lung cancer is initially categorized into two subtypes: non-small cell lung cancer (NSCLC) and small cell lung cancer (SCLC). NSCLC accounts for approximately 85% of all LC patients. Among NSCLC subtypes, adenocarcinoma is the most frequent (40–50% of patients). Squamous cell carcinoma is diagnosed in 20–30% of patients, and large-cell carcinoma in approximately 3% of patients [4]. According to SEER (Surveillance, Epidemiology, and End Results) database, only 21.7% of all patients with lung cancer have an overall survival of 5 years or more after diagnosis [5]. Thanks to significant advances which have been made in the treatment of lung cancer over the past few years, patients diagnosed with metastatic lung cancer and eligible for targeted therapies or immunotherapies are surviving significantly longer. Currently, the 5-year survival rate ranges from 15% to 50% of advanced LC patients, depending on the availability of personalized treatment [6]. The examination of somatic oncogenic driver mutations has become the standard of care in certain NSCLC patients. There is a rapid increase in the availability of approved drugs for NSCLC patients with oncogenic driver alterations that have resulted in the prolongation of progression-free survival (PFS) and overall survival (OS) of patients.

The most frequent oncogenic alterations are *KRAS* (*Kirsten Rat Sarcoma Viral Oncogene Homolog*) gene mutations, which are presented in 30–50% of NSCLC patients. Among them, 12% of patients present G12C (p.Gly12Cys, c.34G>T) *KRAS* gene mutation. *EGFR* (*Epidermal Growth Factor Receptor*) gene mutations are present in 10–40% of patients and are predominantly detected in female patients with adenocarcinoma, in non-smokers, and in Asian patients [7]. Other relatively common driver genetic abnormalities are found in the following oncogenes: *ALK* (*Anaplastic Lymphoma Receptor Tyrosine Kinase*) (4.5% of patients), *MET (MET Proto-Oncogene*, *Receptor Tyrosine Kinase)* (4% of patients), *BRAF* (*B-Raf Proto-Oncogene*, *Serine/Threonine Kinase*) (2.5% of patients), *RET* (*Ret Proto-Oncogene*) (1.5% of patients), *ROS1* (*ROS Proto-Oncogene 1*, *Receptor Tyrosine Kinase*) (1.5% of patients), *NTRK* (*Neurotrophic Receptor Tyrosine Kinase*) (1% of patients) [8]. Molecularly targeted therapies should be used in advanced NSCLC patients with these genetic abnormalities. The oldest and best-known group of molecularly targeted drugs are the EGFR tyrosine kinase inhibitors (TKIs). Three generations of EGFR TKIs are available for patients harboring *EGFR* gene mutations: first-generation TKIs—erlotinib and gefitinib, second-generation TKIs—afatinib and dacomitinib, and third generation TKI—osimertinib. There are also drugs specifically for patients with insertions in exon 20 of the *EGFR* gene (amivantamab, mobocertinib) [8].

Three generations of molecularly targeted drugs are also used in patients with *ALK* gene rearrangements. Rearrangements of the *ALK* gene result in the abnormal expression of constitutively active ALK fusion proteins [9]. In the phase III PROFILE 1014 trial, crizotinib, the first-in-class ALK tyrosine kinase inhibitor, demonstrated improved outcomes compared to platinum-based chemotherapy as the first-line therapy for advanced NSCLC patients with *ALK* gene rearrangement. This trial established first-line ALK tyrosine kinase inhibitor as the standard of care for this patient population [10]. Since then, several new ALK inhibitors have proved to be more effective: ceritinib, alectinib, brigatinib, ensartinib and lorlatinib. The previously mentioned crizotinib was also the first approved TKI for the treatment of *ROS1*-rearranged advanced NSCLC patients. Currently, entrectinib is more often used in patients with *ROS1* gene rearrangement due to the high intracranial efficacy of this drug. These agents are administered as the initial treatment approach, providing effective and targeted therapy for this specific patient population [11,12].

While early studies demonstrated some effectiveness of single-agent BRAF inhibitors in the treatment of NSCLC patients with V600E (p.Val600Glu, c.1799T>A) *BRAF* gene mutation, combining BRAF and MEK (Mitogen-Activated Protein Kinase Kinase 1) inhibitors (dabrafenib with trametinib) has shown even greater efficiency [13,14,15]. In lung cancer, oncogenic *MET* gene activation can happen through two mechanisms: exon 14 skipping mutations or *MET* gene amplification [16]. Capmatinib and tepotinib, which are two types of Ib MET inhibitors, have received regulatory approval for the treatment of patients with exon 14 skipping mutations in the *MET* gene [17]. However, capmatinib has shown potential for the treatment of NSCLC patients with *MET* amplification (≥10 gene copy number in cancer cells) who have previously received immunotherapy and/or platinum-based chemotherapy [18]. Moreover, two RET inhibitors (selpercatinib and pralsetinib) were registered in NSCLC patients with *RET* gene rearrangements, and two NTRK inhibitors (larotrectinib and entrectinib) in patients with *NTRK1*, *NTRK2* or *NTRK3* gene rearrangements. Significantly, these agents have shown high response rates in intracranial and extracranial lesions [19,20,21]. RET and NTRK inhibitors are among the first tissue agnostic drugs that could be used in cancer patients with *RET* and *NTRK* gene rearrangements, regardless of the pathomorphological type of cancer [22].

*KRAS* gene mutations were considered to be “untargetable” until 2021, when the first KRAS inhibitor—sotorasib—was approved for use in NSCLC patients, who were treated with at least one systemic treatment and progressed. Another KRAS inhibitor is adagrasib, which is approved only by the FDA. These drugs are more effective and have a better safety profile than standard therapies (docetaxel). Sotorasib and adagrasib could only be used in patients with the G12C *KRAS* gene mutation. Both molecules bind to cysteine, which is substituted instead of glycine in codon 12 of the *KRAS* gene, thereby inhibiting the hyperactivity of the mutated KRAS protein [23].

## 2. *RAS* Genes Family Structure and Function

The *RAS* (*RAS Proto-Oncogene*, *GTPase*) gene family includes three homologs, each consisting of seven exons, occupying different chromosome *loci*: *KRAS* in *locus* 12p12.1, *NRAS* (*Neuroblastoma RAS Viral (V-Ras) Oncogene Homolog*) in *locus* 1p13.2, and *HRAS* (*Harvey Rat Sarcoma Viral Oncogene Homolog*) in *locus* 11p15.5. The three *RAS* genes encode proteins of 188–89 amino acids that share 82–90% sequence homology [24]. There are three parts to the amino acid chain of the RAS proteins. The first conserved region consists of 85 amino acids. The second part (80 amino acid residues) has 85% homology between the RAS proteins. The third part is a highly variable region with only 8% homology [25].

RAS belongs to the monomeric GTP-binding proteins and is inactive in its GDP-bound form. Proteins from the RAS family belong to the small GTP-binding proteins, containing the “G domain”, which makes them part of the group of molecular switches [25,26]. The proteins undergo conformational changes during the conversion of the guanosine diphosphate (GDP)-bound to the guanosine triphosphate (GTP)-bound state. The RAS protein contains Swich I (amino acids 30–40) and Swich II (amino acids 58–72) regions, which form a spacer for binding effector and regulatory proteins [27]. The protein also contained the P-loop (amino acids 10–14), also known as the Walker A motif, involved in the switch function [27,28]. The lowest homology in the RAS proteins sequence is observed in the HVR (Highly Variable Region) motif located at the C-terminus end, which is responsible for anchoring the protein to the cell membrane. Importantly, activating mutations in *RAS* genes involve the P-loop and Switch II region [27,28].

*RAS* mutations lead to their constant activation and further overactivation of signaling pathways involved in cancer cell division and survival. Activated RAS proteins with attached GTP bind and activate RAF (Raf Proto-Oncogene) serine-threonine kinases. Activated RAF phosphorylate and activate MEK1/2 (Mitogen-Activated Protein Kinase 1/2) kinase. MEK1/2 phosphorylates ERK1/2 (Extracellular Signal-Regulated Kinase) to activate it. The transition of ERK1/2 to the nucleus activates the expression of downstream genes and transmits signals for proliferation [29,30].

Another pathway of action of RAS is the direct binding and activation of PIK3CA (Phosphoinositide-3-Kinase, Catalytic, Alpha Polypeptide). It leads to the activation of the PIK3CA-AKT/mTOR signaling pathway associated with the survival of the cells and proliferation [31,32]. GTPase RAL is the third downstream RAS effector. Studies have shown that different *RAS* mutations selectively activate particular effector pathways in non-small cell lung cancer (NSCLC) [33].

## 3. *KRAS* Gene Mutations Status in Cancers

*KRAS* alterations affect 30–40% of lung cancers, 40–50% of colorectal cancers and 85–90% of pancreatic cancers. These changes are either point mutations occurring at codons 12, 13, 61, 117, or 146, as well as 5- to 50-fold amplification of the gene [34]. Zhu et al. derived data from the AACR GENIE 9.0 public database and showed that *KRAS* mutations in the USA concern 87% of pancreatic ductal adenocarcinoma, 43% of colorectal cancer, and 33% of lung adenocarcinoma patients [35]. Wang et al. indicate the presence of *KRAS* variants in rare cancers. They analyzed using the NGS method 3453 patients including 122 rare tumor subtypes. Authors concluded that soft tissue malignancies (1076 cases), digestive tract cancers (931 cases), nervous system neoplasms (732 cases), cancer of unknown primary (CUP, 262 cases), and respiratory system cancers (207 cancers) are among the five most common types of malignancies affected by mutations in the *KRAS* gene [36]. They found that *KRAS* lesions were identified in 8.7% of all examined patients [36]. *KRAS* mutations included 21 missense mutations, of which G12D (29.2% of patients), G12V (24.6% of patients) and G13D (10.8% of patients) were the most common. Interestingly, G12C was observed in 0.6% of all and 5.7% of lung sarcomatoid carcinoma patients [36]. The G12C variant affects 41% of patients with lung adenocarcinoma with *KRAS* mutations, while the G12D and G12V variants are the two most common variants in colorectal cancer and ductal carcinoma of the pancreas [37].

*KRAS* mutations concern around 35% of lung adenocarcinomas in the United States, and approximately 13% in China [37]. There is an apparent difference in the incidence of *KRAS* mutations between Western and Asian populations—these mutations are three times more common in Caucasian than in Asian NSCLC patients [37,38,39]. The most common *KRAS* mutations are G12C, G12D (p.Gly12Asp, c.35G>A), and G12R (p.Gly12Arg, c.34G>C). Judd et al. tested 17095 NSCLC tumors using NGS, and in 4706 tumors (27.5% of tumors), *KRAS* gene mutations were identified [39]. Among patients with *KRAS* mutations, 40% had G12C variant, 19%—G12V, 15%—G12D, 6%—G12A, 4%—other variants in codon 12, 7%—variants in codon 13, 7%—variants in codon 61, and 2%—other, rare *KRAS* mutations. Smoking is strongly associated with *KRAS* mutations in lung cancer—most patients with these mutations are current or former smokers. These mutations occur in 20–40% of adenocarcinoma patients compared to approximately 5% of squamous-cell lung cancer patients.

Nearly 32% of *KRAS* mutations and 36% of *KRAS* G12C mutations co-occurred with other targetable and non-targetable alterations [36]. Generally, 97% of *KRAS* mutated tumors have other genetic changes. Genetic alterations coexisting with *KRAS* occur in following genes: *CDKN2A* (*Cyclin Dependent Kinase Inhibitor 2A*), *PIK3CA*, *ATM* (*Ataxia-Telangiectasia Mutated Gene)*, *TP53 (Tumor Protein P53)*, *STK11* (*Serine-Threonine Kinase 11*), *KEAP1* (*Kelch-like ECH-associated Protein 1*), *HGFR* (*Hepatocyte Growth Factor Receptor*), and *HER2* (*Erb-B2 Receptor Tyrosine Kinase 2*) [38]. Table 1 shows tumor suppressor genes and their primary functions, in which variants that can be found coexisting with mutations in the *KRAS* gene in patients with NSCLC. Mutations in the *KRAS* gene seldom coexist with abnormalities in the following genes: *EGFR*, *ALK*, *NTRK1/2/3 RET*, and *ROS1* [38,40,41]. Judd et al. indicate that *KRAS*-associated variants are located in genes such as *TP53*, *STK11*, *NF1* (*Neurofibromin 1*), *KEAP1*, *U2AF1* (*U2 Small Nuclear RNA Auxiliary Factor 1*), *CDKN2A* or *ATM* [39]. Authors found a case of coexistence of substitution in codon 61 of the *KRAS* gene and mutations in the *EGFR* gene and cases of coexistence of *BRAF* and *KRAS* mutations [39].

A study by Dong et al. indicated that *TP53* and *KRAS* genes mutation coexistence is a positive predictive factor for immunotherapy effectiveness in non-small cell lung cancer. The study confirmed that *TP53* and *KRAS* co-mutated patients had increased expression of PD-L1 on tumor cells [42]. Tomasini et al. tested 218 patients with NSCLC in any stage with *KRAS* and *TP53* mutations who received chemotherapy [43]. They proved that OS was longer for patients with *TP53* and *KRAS* wild-type NSCLC compared to patients with *KRAS* or *TP53* mutations or double mutant tumors [43]. In the case report of a non-smoking man with squamous cell lung cancer, co-mutations of *TP53* and *KRAS* genes were detected using next-generation sequencing (NGS) technology [44]. Treatment of pembrolizumab in combination with gemcitabine as a rescue therapy was administrated and a significant partial response for more than 7 months was obtained [44]. The authors concluded that both *TP53* and *KRAS* mutations should be considered in advanced squamous cell lung cancer as a potential predictive factor for response to immunotherapy [44].

In the study of Zhao et al., 89 advanced NSCLC (75 adenocarcinomas, 12 squamous cell carcinomas and 2 adenosquamous carcinomas) patients were tested using NGS (Circulating Single-Molecule Amplification and Resequencing Technology). NGS was performed in circulating free DNA (cfDNA) from liquid biopsy (peripheral blood). All patients included in the study received first-line chemotherapy with carboplatin in combination with pemetrexed or paclitaxel. Among them, 50 had *KRAS*, *TP53* or *PIK3CA* mutations [45]. Coexistence of mutations was identified in 17 patients, including *KRAS* (exon 2) and *TP53* mutations in ten cases, *PIK3CA* and *TP53* mutations in four cases, *KRAS* (exon 4) and *PIK3CA* mutations in one case, and *KRAS* and *PIK3CA* with *TP53* in two cases. Patients without mutations had longer PFS than those with mutations in *KRAS*, *TP53*, *KRAS* and *TP53* or *PIK3CA* and *TP53* genes [45]. Mutation coexistence in *KRAS* and *TP53* or *PIK3CA* and *TP53* genes was related to shorter PFSs than those with a single *KRAS* or *TP53* mutation [45]. Further, the authors observed that *KRAS* and *TP53* co-mutation is associated with inflamed tumors and inactivation of *STK11* is associated with the absence of T cells in *KRAS*-mutant tumors [46]. Additionally, Liu et al. observed that the presence of *KRAS* mutations in tumor cells is associated with intensive T lymphocyte infiltration in the tumor microenvironment in NSCLC patients. In addition, *KRAS* mutant tumor cells more often showed high or moderate expression of PD-L1 than wild-type *KRAS* gene tumor cells. NSCLC tumors with *KRAS* mutations had more somatic mutations (high Tumor Mutation Burden, TMB) than tumors without mutations in this gene. Unfortunately, the authors did not provide information on mutations coexisting with mutations in the *KRAS* gene [47].

Benge et al. studied the impact of *KRAS* and *TP53* mutations on outcomes after first-line systemic therapy in patients with metastatic or recurrent NSCLC with *STK11* gene mutations [48]. They tested 1 385 patients with NGS performed in tumor tissue or plasma samples. *STK11* mutations were detected in 77 patients. They included 62 patients from this group in further analyses. Mutations in exons 1–2 of the *STK11* gene were observed in 22 cases, and mutations in exons 3–9 were observed in 40 patients, while the most common mutation in *STK11* was p.L282Afs*3 [48]. In the *STK11*-mutated group, 18 had an *STK11* single mutation, and 44 patients had coexistence STK11 mutations with mutations in the following genes: *KRAS* in 19 patients, *TP53* in 18 patients, and *KRAS* with *TP53* in 7 patients [48]. Patients with *STK11* and *KRAS* co-mutations had a shorter median PFS (2.4 months) compared with patients with *STK11* mutations alone (5.1 months) and *STK11* and *TP53* co-mutations (4.3 months) as well as *STK11* and *KRAS* with *TP53* (13 months) [48]. Moreover, patients with *STK11* and *KRAS* mutation coexistence had shorter median OS (7.1 months) compared with patients with *STK11* mutations alone (16.1 months), *STK11* and *TP53* co-mutations (28.3 months), or *STK11* and *KRAS* with *TP53* co-mutations (22 months).

There are indications that *STK11* and *KEAP1* mutations are significant adverse predictors for the efficacy of immune check point inhibitor (ICIs) therapy in NSCLC patients with *KRAS* mutations [49]. However, the neutrophil–lymphocyte ratio (NLR) is impacted by *STK11* but not by *KEAP1* mutations. It suggests differences in the immunotherapy resistance mechanism connected with the presence of both mutations. The authors concluded that *KRAS* mutations could be associated with improved survival in NSCLC patients treated with immunotherapy in the absence of mutations in the *STK11* and *KEAP1* genes in tumor cells [49].

*KEAP1* is the third most mutated gene in adenocarcinoma of the lung and is often associated with mutations in *KRAS* [50]. As mentioned above, the coexistence of mutations in *STK11*, *KEAP1,* and *KRAS* genes is associated with a lack of benefit from immunotherapy [51]. NSCLC patients with *KRAS* and *STK11* co-mutations are also more likely to carry the mutations in *KEAP1* and *ATM* genes and are predisposed to the development of a cold tumor (without immune cells in tumor microenvironment). The lower number of somatic mutations and reduced activity of anti-inflammatory signaling is also evident in patients with co-mutations in *KRAS* and *KEAP1* or *ATM* genes [39]. Judd et al. indicate that oncogenic variants are observed frequently in patients with *KRAS* and *ATM* mutation coexistence. In this group of patients, mutations in the following genes were shown: *CCND1* (*Cyclin D1*) (3.8% of patients), *FGF3* (*Fibroblast Growth Factor Receptor 3*) (4.2% of patients), *FGF4* (*Fibroblast Growth Factor Receptor 4*) (3.5% of patients). In contrast, mutations in these genes occurred in 0.7–0.8% of patients with *KRAS* gene mutations that did not coexist with mutations in the *ATM* gene [39].

The researchers also pointed out that the *U2AF1* gene is most often mutated in coexistence with *KRAS* mutations than other mutations in codon 12. Moreover, *NF1* variant incidence was more common in patients with *KRAS* mutations in codon 13 compared to patients with other *KRAS* mutation subtypes [39]. The role of mutations of the *U2AF1* gene, especially S34F (p.Ser34Phe, c.101C > T), in NSCLC patients, is not completely understood, but the activity of the gene is strongly related to splicing processes and the mentioned mutations may affect the selection of the 3’ splice site [50]. Furthermore, loss-of-function mutations in the *NF1* gene affect about 11% of all lung adenocarcinomas and occur in about 3% of cases in coexistence with oncogenic *KRAS* gene variants [50].

Genetic abnormalities coexisting with *KRAS* gene mutations may be related to the tumor microenvironment, the capacity of the immune system’s anticancer response, or the efficacy of the treatment: chemotherapy, immunotherapy or even molecularly targeted therapies. For example, it has been shown that the acquisition of *HER2* copy number gain in patients with G12C mutations in the *KRAS* gene may be an important mechanism of resistance to sotorasib in NSCLC patients [52]. Moreover, only 20% of patients with coexisting *KRAS* and *KEAP1* mutations responded to sotorasib therapy. However, 44% of patients with the G12C mutation in the *KRAS* gene and without *KEAP1* mutations achieved partial remission during therapy with this KRAS inhibitor [53]. Inactivating mutations in *NF1* affect the permanent activation of RAS signaling pathways, leading to increased cell division and tumor growth [54]. Currently, it is possible to treat NSCLC patients with molecularly targeted therapies only in the presence of the G12C mutation in the *KRAS* gene. However, the results of treatment may be unsatisfactory. It appears that the molecular and immunological background in *KRAS*-mutated patients may be a key factor in the efficacy of treatment of such patients, both in terms of immunotherapy and molecularly targeted therapy.

## 4. *HRAS (HRas Proto-Oncogene)*, *NRAS* (*NRAS Proto-Oncogene*) Genes Mutations Status in Non-Small Cell Lung Cancer

*HRAS* mutations affect approximately 0.5% of NSCLC patients. These mutations are most common in codon 61 of the *HRAS* gene [55]. Mathiot et al. used the NGS technique to identify the Q61L (p.Gln61Leu, c.182A > T) mutation in the *HRAS* gene in four advanced NSCLC patients (0.25% of 1614 tested tumor) [55]. They found three additional cases with this mutation in the literature. All patients were current or former smokers. Tamiya et al. indicated that the most frequent variants of *NRAS* and *HRAS* genes related to codon 61 (78% of cases with *NRAS* mutations) and codon 13 (80% of cases with HRAS mutations) [56].

Ohashi et al. showed that, among 4562 patients with lung cancers, *NRAS* mutations were detected in 30 (0.7% of patients), and 80% of them had adenocarcinoma histology [57]. *NRAS* mutation is considered a predictive factor for immunotherapy. Dehem et al. conducted a study comparing the effectiveness of chemotherapy, immunotherapy, and chemoimmunotherapy in patients with *NRAS* gene mutations [58]. They enrolled 153 advanced NSCLC patients with *NRAS* mutations (predominantly with adenocarcinoma). Objective response rate (ORR) and median PFS in patients treated with platinum doublet chemotherapy, immunotherapy, and chemoimmunotherapy were as follows: 41% and 5.1 months, 33% and 6.9 months, and 75% and 8.6 months, respectively [58].

## 5. Own Experience Regarding the Frequency of *KRAS* Mutations and Their Impact on the Effectiveness of Immunotherapy in NSCLC Patients

We determined the molecular profile of 126 NSCLC patients (66 males, 60 females, 109 adenocarcinoma patients, 14 squamous cell carcinoma patients, 2 adenosquamous cell carcinoma patients, and 1 patient with NSCLC NOS) using next-generation sequencing technology. The Oncomine Focus Assay on the Ion Torren S5 platform (Thermo Fisher Scientific, USA) was used. *KRAS* mutations occurred in 30.2% of NSCLC patients. *KRAS* mutations were significantly more common in smokers compared to non-smokers (χ2 = 4.74, *p* = 0.029) and occurred with almost identical frequency in men and women. The following mutations in the *KRAS* gene were detected: p.Gly12Cys in twenty-four patients (19.05% of patients), p.Gly12Val in six patients (4.8% of patients), p.Gln61His in three patients (2.4% of patients), p.Gly12Ala in two patients (1.6% of patients), p.Gly13Asp in one patient (0.8% of patients), p.Gln61Leu in one patient (0.8% of patients), and coexistence of p.Gly12Val with p.Gly12Ser mutation in one patient (0.8% of patients).

In the group of 126 patients with a known molecular profile, 68 patients received immunotherapy (the age of the study group was 63 ± 8.9 years, and the group included 31 men and 37 women). Immunotherapy was administered to sixty patients with adenocarcinoma, five patients with squamous cell carcinoma, two patients with adenosquamous carcinoma, and one patient with NSCLC NOS. In the first line of treatment, 14 patients received pembrolizumab in monotherapy, and 30 patients received pembrolizumab in combination with chemotherapy. In the second line of treatment, 15 patients were treated with atezolizumab and 9 patients with nivolumab. *KRAS* mutations were present in 36 patients treated with immunotherapy. The characteristics of the group are included in Table 2.

A partial response to treatment occurred in 16 patients (24.6%), including 7 patients with mutations in the *KRAS* gene (20%), while 31 patients (47.7%) experienced disease stabilization, including 18 patients with *KRAS* mutations (51.4%). These differences were not statistically significant. The median PFS was 6.3 months (95% CI: 4.8–10.7) in patients with mutations in the *KRAS* gene and 5.2 months (95% CI: 4.5–26.5) in patients with wild type of *KRAS* gene (HR = 0.882, 95% CI: 0.463–1.677, *p* = 0.7). Meanwhile, the median overall survival in patients with *KRAS* gene mutations reached 20.8 months (95% CI: 15.7–29.5) and was not reached in patients without mutations in this gene (HR = 1.597, 95% CI: 0.681–3.746, *p* = 0.282). The median PFS and OS calculated from the start of immunotherapy did not depend on the sex or age of patients, smoking status, line of immunotherapy, or *KRAS* gene status.

## 6. Effectiveness of Immunotherapy in *KRAS*-Mutated NSCLC Patients—A Literature Review

**Figure 1:**
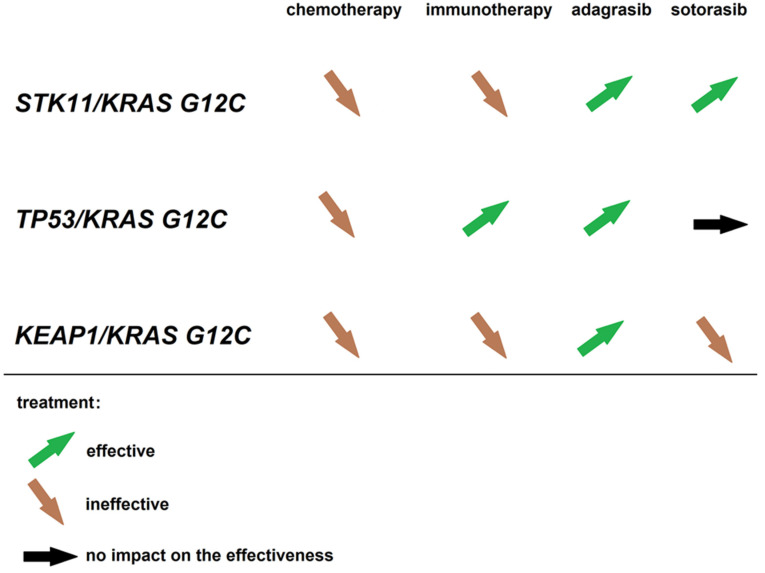
The co-existing mutations in *KRAS* (G12C), *STK11*, *TP53*, and *KEAP1* genes and the efficacy of various therapies in NSCLC [48,49,53,61,62].

Immunotherapy has emerged as a promising treatment modality for non-small cell lung cancer patients, including those with a G12C mutation in the *KRAS* gene. *KRAS* G12C is one of the most common *KRAS* mutations in NSCLC and has been associated with a poor prognosis [59]. On the other hand, immune checkpoint inhibitors such as monoclonal antibodies against PD-1 or PD-L1 have shown efficacy in NSCLC patients, including those with *KRAS* mutations [60]. Figure 1 outlines the impact of co-existing mutations in *KRAS* (G12C), *STK11*, *TP53*, and *KEAP1* genes on the efficacy of various therapies in NSCLC.

The KEYNOTE-042 trial evaluated pembrolizumab as a first-line treatment in NSCLC patients with PD-L1 expression on ≥1% of tumor cells (TC) and showed improved overall survival compared to chemotherapy, regardless of *KRAS* mutations status. The clinical benefit from pembrolizumab over platinum-based chemotherapy noted in the overall population was maintained when outcomes were assessed according to *STK11*, *KEAP1,* and *KRAS* mutational status. In the pembrolizumab arm, patients with *KRAS* G12C had improved ORR and longer PFS and OS compared with those with *KRAS*^WT^ [63].

In addition, several observational studies have been conducted and published. Sciortino et al. carried out a single-center retrospective observational study to find the correlation between response to immunotherapy and *KRAS* mutations presence. The results of this study do not reveal a clear correlation between mutations and response to immunotherapy [64]. Second, a similar single-institution study was performed by Kartolo et al., with close results [65]. *KRAS* mutation status did not have a significant impact on ICI efficacy or safety. However, a nonsignificant trend towards worse survival was noted in patients treated with ICIs whose tumors harbored the *KRAS* G12C variant compared to those with wild-type *KRAS* gene [65]. On the contrary, opposite results were found in a small study group collected by Cefali et al. The authors concluded that *KRAS* mutations can be considered as a predictive marker of prolonged response to first-line ICIs in NSCLC patients with high expression of PD-L1 [66].

Some studies suggested a lower response rate to immunotherapy in *KRAS*-mutated NSCLC patients who had coexisting mutations in *STK11* gene. The investigation by Miralli et al. is one of the smaller studies that addressed whether co-occurring mutations in *KEAP1*, *STK11*, *PBRM1 (Polybromo 1)*, and *SMARCA4 (Mitotic Growth And Transcription Activator)* genes could facilitate the identification of adenocarcinoma patients unresponsive to ICIs. Data indicates that co-occurring mutations of these genes distinguished a subset of patients without immunotherapy benefits [61].

A global multicenter registry focusing on the efficacy of immunotherapy in NSCLC patients with driver genetic alterations was IMMUNOTARGET. In the IMMUNOTARGET registry, *KRAS*-mutated patients experienced the highest ORR (26%) and long-term responses in comparison to those with other molecular driver alterations. The patients with *KRAS* mutations had the lowest rate of rapid progression to ICIs (<2 months) compared to those harboring other oncogenic alterations [67].

Scoulidis et al. made one of the largest observations on the effectiveness of nivolumab and pembrolizumab in NSCLC patients with *KRAS* gene mutations with or without coexisting mutations. These data come from two clinical trials: SU2C and CheckMate 057 [62]. The SU2C study included 54 patients with *KRAS* and *STK11* co-mutations, 56 patients with *KRAS* and *TP53* co-mutations, and 63 patients with *KRAS* mutations and no co-mutations. Response to immunotherapy occurred in 7.4% of patients from the first group, 35.7% of patients from the second group, and 28.6% of patients from the third group. In the CheckMate 057 clinical trial, there was no response to nivolumab in the first group of patients. In the second and third groups, 57.1% and 18.2% of patients achieved response to treatment, respectively. However, in this study, the number of patients in each group was low and amounted to 6, 7, and 11 patients, respectively. In the SU2C study, progression-free survival and overall survival were also assessed depending on the presence of mutations in the *KRAS*, *STK11*, and *TP53* genes. The median PFS was significantly lower in patients with the coexistence of mutations in the *KRAS* and *STK11* genes (1.8 months) compared to patients with the coexistence of mutations in the *KRAS* and *TP53* genes (3 months) or with the mutations in the *KRAS* gene (2.7 months). Similar results were obtained regarding overall survival; the median OS in the analyzed groups was, respectively, 6.4 months, 16 months, and 16 months. The authors concluded that the effectiveness of immunotherapy did not depend on the presence of mutations in the *KRAS* gene, but it was affected by the type of coexisting mutations. It seems that the coexistence of *KRAS* mutations with mutations in the *STK11* gene is a negative predictive factor for immunotherapy, and the coexistence of *KRAS* mutations with mutations in the TP53 gene is a favorable predictive factor for this method of treatment [62].

PD-L1 expression is usually higher in patients with *KRAS* gene mutations compared to patients with *KRAS^wt^*. The lack of PD-L1 expression is found in about 25% of *KRAS^mut^* patients and about 40% of *KRAS*^wt^ patients, while PD-L1 expression on ≥50% of tumor cells is found in about 40% of *KRAS^mut^* patients compared to 20% of *KRAS^wt^* patients. Patients with *KRAS* gene mutations are usually characterized by a higher level of TMB (about 8–10 mutations per Mbp) than patients with *KRAS^wt^* (about 5–6 mutations per Mbp). Patients with *KRAS* gene mutations smoke cigarettes more often than patients with *KRAS^wt^*, which generates more somatic mutations in tumor cells. In turn, a high number of somatic mutations gives rise to development of numerous neoantigens. Tumors with high TMB become highly immunogenic (“hot”, inflammatory tumors). “Hot” tumors are characterized by infiltration from immune cells, primarily cytotoxic T lymphocytes. Less than 10% of patients with *KRAS*^mut^ have “cold” tumors (no CD8^+^ T cell infiltrates). On the other hand, 25% of patients with *KRAS^wt^* may show no infiltration from cytotoxic T lymphocytes within the tumor. At the same time, patients with *KRAS* gene mutations show a lower number of immunosuppressive cells in the tumor (regulatory T cells, M2 macrophages, myeloid-derived suppressor cells) than patients with *KRAS^wt^* [47].

## 7. Effectiveness of KRAS Inhibitors in NSCLC Patients with G12C Mutations in the *KRAS* Gene

Clinical trials have shown propitious results for the use of molecularly targeted therapy in NSCLC patients with *KRAS* G12C mutations. The CodeBreaK 200 trial revealed that sotorasib significantly improved PFS and had a more promising safety profile compared with docetaxel, in patients with advanced *KRAS* G12C NSCLC treated previously with other anticancer drugs. Patients treated with sotorasib (171 patients) had a significantly higher median PFS (5.6 vs. 4.5 months) than those treated with docetaxel (174 patients). Moreover, the percentage of patients remaining progression-free for one year or more was 24.8% in the sotorasib arm and 10.1% in the docetaxel arm [23]. Another KRAS inhibitor is adagarsib which was studied in KRYSTAL-1 clinical trial. A total of 116 *KRAS*^G12C^ NSCLC patients had been treated with adagrasib (median follow-up 12.9 months). Of 112 patients with measurable disease at baseline, 48 (42.9%) had a proven objective response [68]. The median duration of response was 8.5 months, and the median progression-free survival was 6.5 months [68].

However, moving molecularly targeted therapy to the frontline, the efficacy of immunotherapy in patients with *KRAS* G12C mutation cannot be neglected. The rationale for combining KRAS inhibitors and pembrolizumab is based on the complementary mechanisms of action of these drugs. Sotorasib targets the cysteine in KRAS protein in *KRAS* G12C mutated patients, which is associated with immune evasion. Sotorasib may enhance the expression of PD-L1 and make the cancer cells more susceptible to immune checkpoint inhibition. Preclinical studies have shown that combining sotorasib with immune checkpoint inhibitors can lead to enhanced anti-tumor activity compared to these treatments used as monotherapy. Another preclinical study investigated the combination of adagrasib and anti-PD-1 or anti-PD-L1 antibodies in mouse models with *KRAS*-mutant cancer. The combination was found to be more effective than monotherapy, with improved antitumor activity and survival [69]. Sotorasib in combination with pembrolizumab or atezolizumab revealed promising efficacy in NSCLC patients with *KRAS* G12C mutation. According to data presented at the 2022 World Conference on Lung Cancer, across all 58 treated patients, the ORR was 29%, including 2 complete responses and 15 partial responses [70]. The disease control rate was 83%, and the median duration of response was 17.9 months [70]. It is worth noting that responses were similar in patients treated with immunotherapy and patients previously not treated with immunotherapy. Overall survival time in patients treated with sotorasib in combination with a PD-1 inhibitor was 15.7 months [70]. Studies on adagrasib and pembrolizumab combination are ongoing (KRYSTAL-7). The phase 2 clinical trial will evaluate the efficacy and safety of adagrasib in monotherapy and combination with pembrolizumab. There will be three cohorts of advanced or metastatic NSCLC patients with *KRAS* G12C mutations who are candidates for first-line treatment. Patients from the first and second cohorts have PD-L1 expression on <1% of TC and will be randomized to adagrasib monotherapy or adagrasib in combination with pembrolizumab. Patients from the third cohort have PD-L1 expression on ≥1% of TC and they will be treated with adagrasib with pembrolizumab. The phase 3 clinical trial will randomize patients with nonsquamous NSCLC with *KRAS* G12C mutation and expression of PD-L1 on <50% of TC to the first-line setting. Patients will receive adagrasib plus pembrolizumab or pembrolizumab plus chemotherapy [71]. Moreover, studies are underway to enhance the effects of immunotherapy by supporting it with inhibitors of the RAS effector pathway, e.g., rigosertib plus nivolumab for *KRAS^mut^* NSCLC patients who progressed on first-line treatment (ClinicalTrials.gov ID NCT04263090) or phase I/II trial studies for the best dose of selumetinib with durvalumab and tremelimumab in patients with stage IV non-small cell lung cancer (ClinicalTrials.gov ID NCT03581487).

## 8. Conclusions

Despite the growing understanding of immunotherapy and its potential benefits, research into its efficacy for *KRAS*-mutated NSCLC patients is still in its early stages. While several studies have been conducted, the results are mixed, and there is no clear consensus on the effectiveness of immunotherapy in this population. The effectiveness of immunotherapy in NSCLC patients seems to depend not only on the presence of mutations in the *KRAS* gene, but primarily on the coexistence of other mutations, including mutations in the *STK11*, *KEAP1*, and *TP53* genes. There is no data to exclude patients with *KRAS* mutations from immunotherapy. There is only one question about the optimal sequence of the treatment and its combinations. *KRAS* mutation testing in NSCLC patients is becoming a diagnostic standard. The test can be performed with a single-gene test (real-time PCR) after excluding mutations in the *EGFR* gene and rearrangement of the *ALK* and *ROS1* genes, or with multi-gene tests (NGS), which is recommended by scientific societies. The *KRAS* gene examination is performed to qualify for therapy with KRAS inhibitors in the second line of treatment. In our clinic, patients with *KRAS* gene mutations receive immunotherapy or chemoimmunotherapy in the first line of treatment, depending on the status of PD-L1 expression on tumor cells. These patients most often benefit from this type of treatment in the form of permanent stabilization of the disease. Unfortunately, they often develop metastases to the central nervous system (CNS) during the treatment. *KRAS* gene mutations prevail in patients with adenocarcinoma, in the course of which metastases to the CNS are frequent (40–60% of patients). In such a situation, CNS metastases undergo local treatment, most often with radiotherapy, which enables the continuation of first-line therapy (in oligometastatic disease) or the start of second-line therapy (if generalized progression has occurred). In patients with the G12C mutation in the *KRAS* gene, whose disease has progressed after immunotherapy or chemoimmunotherapy, we always consider the possibility of using KRAS inhibitors. Of course, this is possible only in patients with good performance status and without renal, hepatic or bone marrow failure and uncontrolled brain metastases. The effectiveness of chemoimmunotherapy or immunotherapy as well as KRAS inhibitors extends the overall survival of advanced NSCLC patients with the G12C mutation in the *KRAS* gene to 2–3 years. Such treatment effects were impossible to obtain even 2 years ago when KRAS inhibitors were not widely available (only in clinical trials). This type of management has become the new standard in the treatment of NSCLC patients.

The combination of immunotherapy with KRAS inhibitors (sotorasib and adagrasib) should be listed among the most promising methods of combined therapy in advanced NSCLC patients with the G12C mutation in the *KRAS* gene. Overall, while further research is needed, immunotherapy has shown promise in NSCLC patients with *KRAS* mutations and may provide an effective treatment option for this patient population.

## Figures and Tables

**Table 1:** Tumor suppressor genes and their primary functions, in which variants can be found coexisting with mutations in the *KRAS* gene in patients with NSCLC.

The Function of Normal Protein	Genes in Which Mutations Occur in Co-Occurrence with *KRAS^mut^*	Mutation Result
Participates in maintaining cell polarity, inhibits cell division	*STK11*	Apoptosis inhibition
Arrests the cell cycle in the case of DNA damage	*TP53*	Cell cycle progression
Involved in the oxidative stress response	*KEAP1*	Cell survival and proliferation
Regulates cell differentiation and division	*NF1*	
Induces apoptosis, arrests the cell cycle	*ATM*	

**Table 2:** Demographic, clinicopathological, and outcome parameters for patient groups with/without *KRAS* mutation.

NSCLC Patients with Known Molecular Profiles Treated with Immunotherapy *n* = 68	*KRAS* *n* (%)	
	*KRAS^mut^ n* = 36	*KRAS^wt^ n* = 32
Age (median 63 years)		
≤63, *n* = 36	20 (55.5)	16 (44.5)
>63, *n* = 32	16 (50.0)	16 (50.0)
Gender		
Males, *n* = 31	17 (54.8)	14 (45.2)
Females, *n* = 37	19 (51.3)	18 (48.7)
Histopathological diagnosis		
Adenocarcinoma, *n* = 60	34 (56.7)	26 (43.3)
Squamous cell carcinoma, *n* = 5	0 (0)	5 (100)
Adenosquamous cell carcinoma, *n* = 2	2 (100)	0 (0)
NSCLC NOS, *n* = 1	0 (0)	1 (100)
Smoking status		
Smoker, *n* = 49	31 (63.3)	18 (36.7)
Non-smoker, *n* = 19	5 (26.3)	14 (73.7)
Immunotherapy		
First line, *n* = 44	18 (42.6)	26 (59.1)
Second line, *n* = 24	18 (75.0)	6 (25.0)
Response to treatment		
PR, *n* = 16	7 (43.75)	9 (56.25)
SD, *n* = 31	18 (58.1)	13 (41.9)
PD, *n* = 21	11 (52.4)	10 (47.6)

## Data Availability

The data presented in this study are available in this article.
